# Complementary therapies in the control of male lower urinary tract symptoms: A systematic review

**DOI:** 10.1590/1518-8345.5897.3543

**Published:** 2022-07-15

**Authors:** Luciana Regina Ferreira da Mata, Paula Giuliana Rodrigues Motter, Cissa Azevedo, Mariana Ferreira Vaz Gontijo Bernardes, Tânia Couto Machado Chianca, Christiane Inocêncio Vasques

**Affiliations:** 1 Universidade Federal de Minas Gerais, Escola de Enfermagem, Belo Horizonte, MG, Brasil.; 2 Universidade Federal de Viçosa, Departamento de Medicina e Enfermagem, Viçosa, MG, Brasil.; 3 Universidade de Brasília, Faculdade de Ciências da Saúde, Brasília, DF, Brasil.

**Keywords:** Complementary Therapies, Lower Urinary Tract Symptoms, Systematic Review, Men’s Health, Phytotherapy, Electroacupuncture, Terapias Complementares, Sintomas do Trato Urinário Inferior, Revisão Sistemática, Saúde do Homem, Fitoterapia, Eletroacupuntura, Terapias Complementarias, Síntomas del Sistema Urinario Inferior, Revisión Sistemática, Salud del Hombre, Fitoterapia, Electroacupuntura

## Abstract

**Objective:**

to evaluate diverse scientific evidence on the effectiveness of complementary therapies in the control of lower urinary tract symptoms in the adult and aged male population.

**Method:**

a systematic review developed according to the PRISMA checklist. The search was performed in the CINAHL, *Embase*, LILACS, PEDro, PubMed, Web of Science and Google Scholar databases.

**Results:**

a total of 585 records were identified and 12 clinical trials were selected that met the inclusion criteria. The outcomes considered by the studies for analyzing effectiveness of the complementary therapies were validated questionnaires to assess the severity of the lower urinary tract symptoms (sensation of incomplete bladder emptying, frequent urination, intermittent flow, weak flow, pain or difficulty urinating, nocturia and urgency) and urodynamics parameters. The studies analyzed the complementary phytotherapy (n=8) and electroacupuncture (n=4) therapies. Six studies related to phytotherapy showed statistical significance. Electroacupuncture showed a significant improvement in the symptoms in two studies.

**Conclusion:**

pytotherapy was effective to control the simptoms related to frequency, urgency, nocturia, incomplete emptying, intermittence, weak flow and effort to initiate urination. To confirm the effectiveness of electroacupuncture, research studies with well-designed methodologies will also be necessary to resolve the divergences between the studies of this review.

## Resumo

**Objetivo:**

avaliar as evidências científicas sobre a efetividade das terapias complementares no controle de sintomas do trato urinário inferior na população masculina adulta e idosa.

**Método:**

revisão sistemática desenvolvida de acordo com o *checklist* PRISMA. A busca foi realizada nas bases de dados CINAHL, *Embase*, LILACS, PEDro, PubMed, *Web of Science* e *Google Scholar*.

**Resultados:**

foram identificados 585 registros e selecionados 12 ensaios clínicos que atenderam aos critérios de inclusão. Os desfechos considerados pelos estudos para analisar a efetividade das terapias complementares foram questionários validados de avaliação da gravidade dos sintomas do trato urinário inferior (sensação de esvaziamento incompleto da bexiga, micções frequentes, fluxo intermitente, fluxo fraco, dor ou dificuldade na micção, noctúria e urgência) e parâmetros da urodinâmica. Os estudos analisaram as terapias complementares fitoterapia (n=8) e eletroacupuntura (n=4). Seis estudos relacionados à fitoterapia mostraram significância estatística. A eletroacupuntura mostrou melhora significativa dos sintomas em dois estudos.

**Conclusão:**

a fitoterapia foi efetiva para controle dos sintomas frequência, urgência, noctúria, esvaziamento incompleto, intermitência, fluxo fraco e esforço para iniciar a micção. Para confirmação da efetividade da eletroacupuntura ainda serão necessárias pesquisas com metodologias bem delineadas para sanar as divergências entre os estudos desta revisão.

## Resumen

**Objetivo:**

evaluar la evidencia científica sobre la efectividad de las terapias complementarias para el control de los síntomas del tracto urinario inferior en la población masculina adulta y adulta mayor.

**Método:**

revisión sistemática desarrollada según la *checklist* PRISMA. La búsqueda se realizó en las bases de datos CINAHL, *Embase,* LILACS, PEDro, PubMed, *Web of Science* y *Google Scholar*.

**Resultados:**

se identificaron 585 registros y se seleccionaron 12 ensayos clínicos que cumplían con los criterios de inclusión. Los resultados que los estudios consideraron para analizar la efectividad de las terapias complementarias fueron cuestionarios validados que evaluaban la gravedad de los síntomas del tracto urinario inferior (sensación de vaciado incompleto de la vejiga, micción frecuente, flujo intermitente, flujo débil, dolor o dificultad para orinar, nicturia y urgencia) y parámetros urodinámicos. Los estudios analizaron las terapias complementarias fitoterapia (n=8) y electroacupuntura (n=4). Seis estudios relacionados con la fitoterapia demostraron significación estadística. La electroacupuntura demostró una mejoría significativa de los síntomas en dos estudios.

**Conclusión:**

la fitoterapia fue efectiva para controlar los síntomas de frecuencia, urgencia, nicturia, vaciado incompleto, intermitencia, flujo débil y esfuerzo para iniciar la micción. Para confirmar la efectividad de la electroacupuntura, aún es necesario que se realicen investigaciones con metodologías bien diseñadas para resolver las diferencias entre los estudios de esta revisión.

**Highlights**

(1) Complementary therapies can be effective in male urinary symptoms.

(2) Phytotherapy was effective in six of the eight studies included.

(3) Studies with electroacupuncture and more robust methodologies are needed.

## Introduction

According to the International Continence Society (ICS), lower urinary tract symptoms (LUTS) can be categorized according to the urination phase: storage, emptying and post-urination^1^. Storage LUTS, such as nocturia and urinary incontinence, are the most reported by the general population, followed by post-urination drip, reduction in urinary flow and sensation of incomplete bladder emptying, which are also common complaints^2^.

LUTS are more frequent with advancing age^3^. In men there are different clinical presentations of LUTS, which are related to the bladder, prostate, urethra, pelvic floor and/or adjacent pelvic organs^1^. Epidemiological studies conducted with the adult and aged male population indicate that prevalence can range from 60% to 84%^4^ ^-^ ^5^. The etiology most frequently associated with LUTS occurrence in men is benign prostatic hyperplasia (BPH)^3^. Although it is not a morbidity that determines severity associated with mortality, these symptoms exert negative impacts on the daily lives of their patients, as they affect quality of life, cause sexual dissatisfaction and increase the risk of depressive disorders^6^ ^-^ ^7^.

There is a variety of treatment strategies for controlling LUTS^8^. The conventional treatment usually involves behavior changes, such as reduced caffeine and alcohol consumption, increased physical activity, and reduced body weight. In some cases, such treatment can be associated with pharmacological measures, with an emphasis on alpha-blockers and 5-alpha-reductase inhibitors^9^. However, there is a percentage of men whose response to the conventional treatment is unsatisfactory and who end up requiring surgical measures^10^. Both options imply medical costs and possible harms associated with adverse effects of the medications or sequelae after invasive interventions^10^.

In this context, interventions based on complementary therapies (CTs) can be an effective strategy to control LUTS in men, especially because they are of lower cost and with minimal adverse effects. The CTs, also called Traditional and Complementary Medicine, are a set of knowledge, skills and practices that originate from the experiences and beliefs of different cultures that complement conventional medicine practices^11^. 

Phytotherapy and acupuncture have been evaluated individually through systematic reviews regarding their effectiveness^12^ ^-^ ^13^ and compared to the conventional treatments to control LUTS^14^ ^-^ ^15^. Nevertheless, it is observed that CTs indication often occur empirically^16^. Therefore, it is expected that a systematic review will be able to summarize the available evidence on the effectiveness of different CTs to control LUTS in the male population and thus favor its implementation in the clinical practice.

Considering popularization of the CTs, there is a need to develop a systematic review that aims at evaluating the diverse scientific evidence on the effectiveness of complementary therapies in the control of lower urinary tract symptoms in the adult and aged male population.

## Method

### Study design

This is a systematic literature review, registered in the International Prospective Register of Systematic Reviews (PROSPERO) platform (Registration number: CRD42021226480), and developed according to the recommendations of the Preferred Reporting Items for Systematic reviews and Meta-Analyses (PRISMA) checklist^17^ to report systematic reviews. 

### Selection criteria

The PICO strategy was used to establish the guiding question, in which the letter P refers to the population or group of patients (men with LUTS), I to the intervention (CTs), C to the comparison (comparison with other interventions) and O to the outcomes (LUTS control). Therefore, this systematic review sought to answer the following question: How effective are CTs in controlling LUTS in the male population?

Only clinical trials evaluating the use of CTs to control LUTS in adult men (18 years old or more) were eligible for the systematic review. There were no restrictions on eligibility regarding language or year of publication.

### Sample definition and period

By reading the titles and abstracts, studies with samples consisting of mixed populations, children and animals were excluded, as well as original research studies whose design was not clinical trial; publications such as reviews, letters, editorials, protocols and case reports; and records that did not have an online summary available. From the full reading, clinical trials that associated conventional treatment with CTs in the intervention group and publications of the clinical trial protocol type were excluded. It is also noted that studies which, although eligible in terms of their titles and abstracts, were not located in full online or made available by the corresponding authors, through email contact.

The search was carried out in the following databases: Index to Nursing & Allied Health Literature (CINAHL) via the CAPES Journals Portal, Embase, *Literatura Latino-Americana e do Caribe em Ciências da Saúde* (LILACS), Physiotherapy Evidence Database (PEDro), PubMed, and Web of Science. The studies were identified from search strategies specifically adapted to each of the databases using terminologies defined by the Medical Subject Headings (MeSH/PubMed) and the Descriptors in Health Science (*Descritores em Ciência da Saúde*, DeCS), as presented in Figure 1. The searches in the databases were conducted in May 2021. The researchers manually searched the reference lists of the studies selected to identify possible references that were missed in the electronic search. Finally, Google Scholar was also considered as a complementary search method.

**Figure 1:** Search strategy and results in each database. Belo Horizonte, MG, Brazil, 2021

Databases	Search strategy
CINAHL	*((“Complementary Therapies” OR “Alternative Medicine” OR “Alternative Therapies” OR “Complementary Medicine” OR “Complementary and Integrative Practices” OR “Medicine, Chinese Traditional” OR Acupressure OR Auriculotherapy OR “Acupuncture, Ear” OR “Acupuncture Therapy” OR “Ear, external” OR Electroacupuncture OR “Acupuncture Points” OR Moxibustion OR Reflexotherapy OR “Foot reflexology” OR “Zone Therapy” OR Meridians) AND (“Urinary Incontinence” OR “Urination Disorders” OR “Urinary Dysfunction” OR “Urinary Symptoms” OR “Urinary Incontinence, Stress”))*
Embase	*(‘lower urinary tract symptom’/exp OR ‘lower urinary tract symptom’) AND (‘chinese medicine’/exp OR ‘chinese medicine’)*
LILACS	*(tw:(((“Terapias Complementares” OR “Medicina Alternativa” OR “Medicina Complementar” OR “Terapias Alternativas” OR “Práticas Integrativas e Complementares” OR auriculoterapia OR “Acupuntura Auricular” OR “Terapia por Acupuntura” OR eletroacupuntura OR “Pontos de Acupuntura” OR moxibustão OR Medicina Tradicional Chinesa OR Meridianos OR Acupressão OR Orelha Externa OR “Complementary Therapies” OR “Alternative Medicine” OR “Alternative Therapies” OR “Complementary Medicine” OR “Complementary and Integrative Practices” OR auriculotherapy OR “Acupuncture, Ear” OR “Acupuncture Therapy” OR electroacupuncture OR “Acupuncture Points” OR moxibustion OR “Medicine, chinese traditional” OR Meridians OR Acupressure OR “Ear, external”)))) AND (tw:(((“Incontinência Urinária” OR “Transtornos Urinários” OR “Incontinência Urinária por Estresse” OR “Urinary Incontinence” OR “Urination Disorders” OR “Urinary incontinence, stress”))))*
PEDro	*Therapy: acupuncture; Problem: incontinence*
PubMed	*((“Complementary Therapies” OR “Alternative Medicine” OR “Alternative Therapies” OR “Complementary Medicine” OR “Complementary and Integrative Practices” OR “Medicine, Chinese Traditional” OR Acupressure OR Auriculotherapy OR “Acupuncture, Ear” OR “Acupuncture Therapy” OR “Ear, external” OR Electroacupuncture OR “Acupuncture Points” OR Moxibustion OR Reflexotherapy OR “Foot reflexology” OR “Zone Therapy” OR Meridians) AND (“Urinary Incontinence” OR “Urination Disorders” OR “Urinary Dysfunction” OR “Urinary Symptoms” OR “Urinary Incontinence, Stress”))*
Web of Science	*((“Complementary Therapies” OR “Alternative Medicine” OR “Alternative Therapies” OR “Complementary Medicine” OR “Complementary and Integrative Practices” OR “Medicine, Chinese Traditional” OR Acupressure OR Auriculotherapy OR “Acupuncture, Ear” OR “Acupuncture Therapy” OR “Ear, external” OR Electroacupuncture OR “Acupuncture Points” OR Moxibustion OR Reflexotherapy OR “Foot reflexology” OR “Zone Therapy” OR Meridians) AND (“Urinary Incontinence” OR “Urination Disorders” OR “Urinary Dysfunction” OR “Urinary Symptoms” OR “Urinary Incontinence, Stress”))*
Google Scholar	*Advanced search With all the words: Complementary Alternative Medicine With the exact phrase: Lower urinary tract symptomsWithout the words: Female; Children*

### Data collection

The web version of EndNoteBasic^®^ was used to groupe the searches and exclude the duplicates. Subsequently, all the references underwent a manual screening to extract key information from the studies (authors; year; country; CTs; objective; sample size; characteristics of the sample; characteristics of the intervention and control groups; treatment and follow-up time; assessment instruments and other outcomes; and conclusions), which were transcribed and organized into a Microsoft Excel spreadsheet that facilitated development of the selection stage.

Selection of the studies was carried out by two researchers, PhD students in Nursing (P1 and P2), independently and in two phases. In the first phase, from the reading of titles and abstracts, those studies potentially eligible for systematic review were identified, considering the inclusion and exclusion criteria defined. In the second phase, the studies selected were read in full and those that did not meet the inclusion criteria were excluded. The selection divergences between both researchers (P1 and P2) were discussed and, when there was no consensus, they were evaluated by a third researcher, PhD in Nursing (P3), who decided on the inclusion or exclusion of the studies.

Two researchers (P1 and P2) independently extracted the relevant data from the articles selected to a Microsoft Excel spreadsheet. The extracted data were as follows: general characteristics of the study (author, year and country of publication), objectives, sample characteristics (number of participants and mean age), characteristics of the intervention [phytotherapy (dose and duration)], acupuncture/electroacupuncture (acupuncture points, manipulation procedures, electrostimulation frequency and intensity, duration and number of sessions), other arms of the study, results (main outcomes analyzed and instruments used) and the conclusion. A third reviewer (P3) evaluated the accuracy of the data collected.

### Data analysis

The quality evaluation of the studies selected was carried out based on the Joanna Briggs Institute (JBI) Critical Appraisal Tool - Checklist for randomized clinical trials. This is an instrument that evaluates the methodological quality and the approach of possible bias in the design, conduction and analysis of data from randomized clinical trials. This checklist consists of 13 questions with four answer options (yes, no, uncertain or not applied)^18^.

In relation to the categorization of the methodological quality of the clinical trials from the instrument applied, studies that had 70% or more “yes” answers were classified as with low risk of bias, with 50% to 69% of “yes” as with moderate risk, and with 49% or fewer “yes” answers as with high risk. 

The risk of bias assessment was performed independently by two researchers (P1 and P2). A third researcher (P3) was considered for evaluating possible divergences. 

## Results

A total of 585 records were identified in the searches conducted in the databases, among which 96 duplicates were removed. After reading titles and abstracts, 478 records were excluded in the first phase and 11 articles were selected for full reading. In addition to these, the search using other methods resulted in the selection of three articles, and another four articles were selected from the reference lists. 

**Figure 2:**
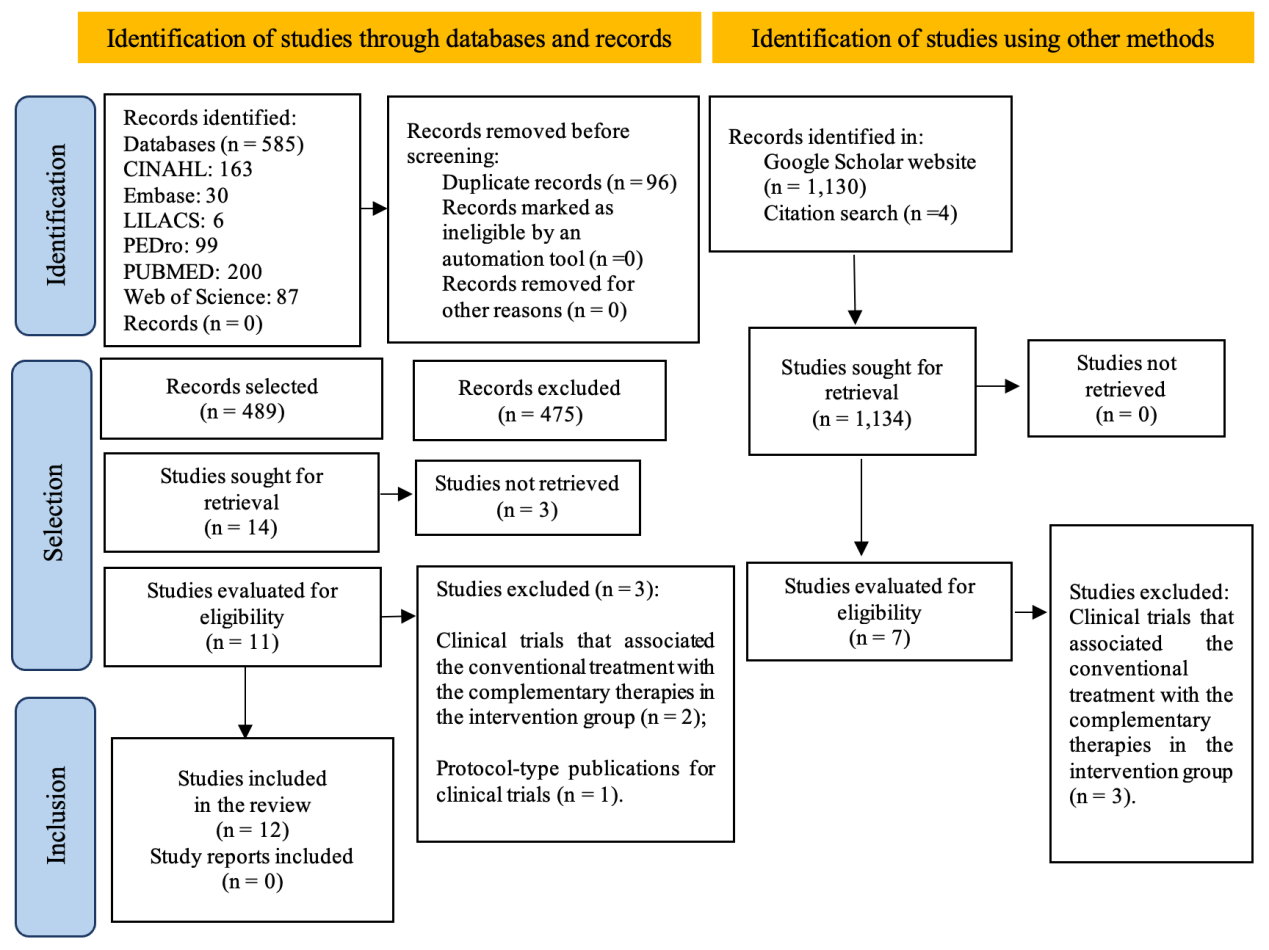
Flowchart corresponding to the searches in the databases and to the selection criteria

Considering the inclusion and exclusion criteria established, of the 18 articles selected and read in full during the second phase, six were excluded. The final sample consisted of 12 studies that met the selection criteria. The process for identification, inclusion and exclusion of the studies is described in Figure 2.

All the studies were published in English between 2001 and 2019. As for the country of origin, four studies were carried out in the United States^19^ ^-^ ^22^, two in Japan^23^ ^-^ ^24^, two in China^25^ ^-^ ^26^, two in the Czech Republic^27^ ^-^ ^28^, one in Italy^29^ and one in Taiwan^30^. 

Most of the clinical trials evaluated phytotherapy (n=8) to control LUTS in the male population^19^ ^,^ ^21^ ^-^ ^24^ ^,^ ^26^ ^-^ ^28^. The other clinical trials in this review addressed electroacupuncture (n=4)^20^ ^,^ ^25^ ^,^ ^29^ ^-^ ^30^. The description of each article is detailed in Figure 3.

**Figure 3:** Description of the characteristics of the studies included in the systematic review (n=12)

Characteristics of the study			Description of the sample		Method applied			Outcomes analyzed		Final conclusions
Author(s) (Year); Country	CT^¶¶^	Objectives	Size(n)	Characteristics (LUTS^‡‡‡^ baseline; mean age ± standard deviation)	Intervention (IG^||^)	Control (CG^§^)	Duration (months)	Assessment questionnaires	Urodynamics parameters	
Gerber, et al. (2001)^19^; USA	PhT^‡^	To evaluate the effects of *Saw palmetto* (*S. palmetto*) extract on urinary symptoms, sexual function, and urinary flow rate in men with LUTS^‡‡‡^.	n: 85 IG^||^: 41 CG^§^: 44	I-PSS^††^ score > 8 without history of prostate surgery; 64.5 (±9.9) years old (IG^||^); 65.3 (±9.7) years old (CG^§^).	160 mg *S. palmetto* capsule (distributed by Nutraceutical), 2x/day (320 mg a day).	Placebo capsule (extra virgin olive oil, gelatin and glycerin), 2x/day.	6	I-PSS^††^	Qmax^||||^	IG^||^ had a mean reduction (improvement) of 4.4 (±5.9) points in the I-PSS score^††^, showing a significant difference (*p* = 0.038) when compared to the CG^§^. Qmax^||||^ improved slightly in both groups, but the changes were not significant (*p* = 0.75).
Bent, et al. (2006)^21^; USA	PhT^‡^	To determine the efficacy of *S. palmetto* extract for the treatment of BPH^¶^.	n: 225 IG^||^: 112 CG^§^: 113	BPH determined by AUASI* score > 8, without history of prostate surgery; 62.9 (±8.7) years old (IG^||^); 63.0 (±7.4) years old (CG^§^).	160 mg *S. palmetto* capsule (distributed by Rexall-Sundown, Inc), 2x/day (320 mg a day).	Placebo capsule (polyethylene glycol 400, bitter liquid with oily aspect without free fatty acids and brown dye), 2x/day.	12	AUASI*	Qmax^||||||^, prostatic volume and PURV	Slight reduction (improvement) in the AUASI* score, 0.72 (±0.35) points in the CG^§^ and 0.68 (±0.35) points in the IG^||^, but without significant difference between the groups (95% CI**: -0.93 to 1.01). Changes were not observed in Qmax^||||^, prostatic volume or PURV.
Barry, et al. (2011)^22^; United States of America	PhT^‡^	To determine the effect of *S. palmetto* extract, up to three times the standard dose, in LUTS^‡‡‡^ attributed to BPH^¶^.	n: 357 IG^||^: 176 CG^§^: 181	AUASI* score between 8 and 24, without history of prostate surgery; 61.3 ± 8.7 years old (IG^||^); 60.7 ± 8.1 years old (CG^§^).	320 mg *S. palmetto* capsule (distributed by Rottapharm). Progressive increase in dosage: -1 capsule/day from the 1^st^ to the 23^rd^ week (320 mg a day); -2 capsules/day from the 24^th^ to the 48^th^week (640 mg a day); -3 capsules/day from the 48^th^ to the 72^th^week (960 mg a day).	Placebo capsules (375 mg polyethylene glycol, 25 mg glycerol and 75 mg gelatin) following a progressive increase in the number of capsules as in the IG.	18	AUASI*	Qmax^||||^ and PURV^||||||^	The mean AUASI* score decreased (improved) by 2.99 points (95% CI**: -3.81 to -2.17) in the CG and by 2.20 points (95% CI: -3.04 to -0.36) in the IG^||^, a difference of 0.79 points between the two groups in favor of the CG^§^. The dose-response comparison showed that there was no improvement in the IG^||^ in any of the doses when compared to the CG^§^. Changes were not observed in Qmax and PURV^||||||^.
Ye, et al. (2019)^26^; China	PhT^‡^	To assess the efficacy and safety of *S. palmetto* among patients with LUTS^‡‡‡^/ BPH^¶^.	n: 325 IG^||^: 159 CG^§^: 166	BPH^¶^ with I-PSS score < 19; 61.5 (±5.2) years old (IG^||^); 60.3 (±6.0) years old (CG^§^).	160 mg *S. palmetto* capsule (distributed by Tad Pharma GmbH)*,* 2x/day (320 mg a day).	Placebo capsule (composition not described), 2x/day.	6	I-PSS^††^	Qmax^‡‡^, prostate volume and urination frequency	The I-PSS^††^ score decreased (improved) in both groups, but the reduction was significantly greater after 24 weeks in the IG^||^ (*p* < 0.001). The IG ^||^ presented a significant increase (improvement) in Qmax^‡‡^ from the 4^th^ week (*p* = 0.011) to the 24^th^ week (p < 0.001). Changes were not observed in prostate volume or urinary frequency.
Noguchi, et al. (2008)^23^; Japan	PhT^‡^	To evaluate the safety and efficacy of the *Ganoderma lucidum (G. lucidum)* extract in men with lower urinary tract symptoms from a dose-escalation study.	n: 50 IG^||^/0.6: 12 IG^||^/6: 12 IG^||^/60: 14 CG^§^: 12	I-PSS^††^ score > 5 without history of prostate surgery; 59.1 (51-70) years old (IG^||^/0.6); 59.2 (50-72) years old (IG^||^/6); 59.4 (50-70) years old (IG^||^/60); 59.7 (50-67) years old (CG^§^).	IG^||^/0.6: 0.6 mg *G.* *Lucidum* tablet, 1x/day; IG^||^/6: 6 mg *G. Lucidum* tablet, 1x/day; IG^||^/60: 60 mg *G.* *Lucidum* tablet, 1x/day. (All tablets distributed by Chlorella Industry).	Placebo tablet (83.75% maltitol, 10% corn starch, 3% vitamin C, 0.2% yellow gardenia and 3% sucrose fatty acid ester), 1x/day.	2	I-PSS^††^	Qmax^||||^, prostatic volume and PURV^||||||^	In the 4^th^ week, the mean change in the I-PSS^††^ score was significantly higher in IG^||^/60, when compared to the CG^§^ (*p* = 0.012) and to the IG^||^/0.6 (p < 0.001). In the 8^th^ week, the mean change in IG^||^/0.6 was significantly lower than in IG^||^/6 (*p* = 0.016) and IG^||^/60 (p = 0.005). Slight improvement of Qmax^||||^, but without significant differences between the groups. No changes were observed in prostatic volume or PURV^||||||^.
Noguchi, et al. (2008)^24^; Japan	PhT^‡^	To evaluate the safety and efficacy in men with mild to moderate lower urinary tract symptoms of the *G. Lucidum* extract, which showed the highest 5α-reductase inhibitory activity among the extracts of 19 edible and medicinal mushrooms.	n: 88 IG^||^_:_ 44 CG^§^: 44	I-PSS^††^ score between 5 and 19, with no history of prostate surgery; 64.0 (±6.9) years old (IG^||^); 64.0 (±8.0) years old (CG^§^).	2 tablets with 3 mg of *G. Lucidum* (distributed by Chlorella Industry), 1x/day (6 mg a day).	2 placebo tablets (83.75% maltitol, 10% corn starch, 3% vitamin C, 0.2% yellow gardenia and 3% sucrose fatty acid ester), 1x/day.	3	I-PSS^††^	Qmax^||||^, Qave^‡‡^, prostate volume and PURV^||||||^	The I-PSS^††^ score decreased (improved) more markedly in the IG^||^ up to the 12^th^ week, showing a significant difference (*p* < 0.001) in the mean change in the score between the two groups. Qmax^||||^ (*p* = 0.400) and Qave^‡‡^ (*p* = 0.080) showed an increase (improvement) in the IG^||^ at the 4^th^ and 8^th^ weeks, but with no significant difference between the groups at the 12^th^ week. Changes were not observed in prostate volume or PURV^||||||^.
Vidlar, et al. (2010)^27^; Czech Republic	PhT^‡^	To evaluate the efficacy and tolerance of cranberry powder in men with lower urinary tract symptoms, elevated PSA^§§^, BPH^¶^ and chronic nonbacterial prostatitis.	n: 42 IG^||^: 21 CG^§^: 21	Histological findings of nonbacterial, acute or chronic prostatitis; 62.0 (±5.4) years old (IG^||^); 64.0 (±5.4) years old (CG^§^);	500 mg cranberry powder capsule (distributed by Decas Botanical Synergies). 3x/day, (1,500 mg a day).	Placebo capsule (composition not described), 3x/day.	6	I-PSS^††^	Qmax, Qave and PURV^||||||^	I-PSS^††^ score significantly lower (better) in the IG^||^ when compared to the CG^§^ (*p* < 0.050). Significant improvement (p < 0.050) of Qmax^||||^, Qave^‡‡^ and PURV^||||||^ in at least 70% of the IG participants^||^, and significant worsening (p < 0.050) of PURV^||||||^ in the CG^§^.
Vidlar, et al. (2015)^28^; Czech Republic	PhT^‡^	To evaluate the effect of cranberry in men with moderate to severe lower urinary tract symptoms.	n: 122 IG^||^/500: 38 IG^||^/250: 43 CG^§^: 41	I-PSS^††^ score > 8 without history of prostate surgery; 52.5 (±5.4) years old (IG^||^/500); 53.3 (±5.2) years old (IG^||^/250); 54.0 (±5.1) years old (CG^§^).	IG^||^/500: 2 x 250 mg cranberry capsules 1x/day (500 mg a day); IG^||^/250: 1 250 mg cranberry capsule and 1 placebo capsule, 1x/day (250 mg a day); (all capsules distributed by Decas Botanical Synergies).	2 placebo capsules (low density maltodextrin, canola oil, sodium aluminum silicate, lake red 40 and lake blue 1), 1x/day.	6	I-PSS^††^	Qmax^||||^, Qave^‡‡^, PURV^||||||^ and bladder emptying volume	Significant reductions (improvement) in the I-PSS^††^ score of IG^||^/500 and IG^||^/250, of -4.1 (±1.9) (*p* < 0.001) and -3.1 (±3.0) (*p* = 0.050) points respectively. Qmax^||||^ (*p* = 0.018), Qave^‡‡^ (*p* = 0.040), PURV^||||||^ (*p* = 0.027) and bladder emptying volume (*p* = 0.014) significantly improved in IG^||^/500.
Johnstone, et al. (2003)^20^; United States of America	EA^†^	To assess the response of lower urinary tract symptoms and PSA^§§^ to EA^†^ in a population of patients with a negative biopsy for prostate cancer.	n: 30 (It does not specify the sample in each study group)	I-PSS^††^ score > 8; 60.7 (±8.2) years old (IG^||^); 64.7 (±5.2) years old (CG^§^/EA^†^ placebo); 63.5 (±3.4) years old (CG^§^/observation).	AcPts***: R10, B40, B32 and B10. 4-5 Hz electrostimulation on R10 and B40. Sessions 3x/week in the 1^st^ and 2^nd^ weeks, and later 1x/week in the 3^rd^, 4^th^ and 8^th^ weeks, 20 min/session.	CG^§^/EA^†^ placebo: insertion of 5 needles in the posterior region of the shoulders, areas unrelated to AcPts*** and without electrostimulation. CG^§^/observation: no intervention during the study.	3 (9 sessions)	I-PSS^††^	Not evaluated	No changes were observed in the I-PSS^††^ score (*p* = 0.063).
Ricci, et al. (2004)^29^; Italy	EA^†^	To assess whether EA^†^ in reflex therapy/acupuncture is able to treat the sensory irritating components of lower urinary tract symptoms that persist after TURP^†††^	n: 42 IG^||^:13 CG^§^/ placebo: 14 CG^§^/ conv: 15	Persistence of sensory irritative symptoms of the lower urinary tract after TURP^†††^; 64.76 years old (52-78 years old).	Somatic AcPts: Conception vessel: 1, 2, 4, 5; Bladder 21, 23 and 32; Auricular AcPts***: prostate and external genitalia. 5-10 Hz electrostimulation, with greater intensity tolerated by the patient. 3 sessions/week, plus maintenance sessions every 15 days, starting from the 4^th^ week, 20 min/session.	CG^§^/placebo: placebo tablet (it does not define composition, number of tablets, or repetitions *per* day); CG^§^/conv: Oxybutynin 5 mg tablet, 2x/day (10 mg a day).	3 (12 sessions)	I-PSS^††^	Qmax^||||^, prostate volume, urination frequency and nocturia	The I-PSS score^††^ decreased (improved) in the CG^§^ and IG^||^ during the first 3 months, but the difference was only significant in the IG^||^ (*p*<0.001). Changes were not observed in Qmax^||||^ and prostate volume. Reductions in daytime urination frequency of 20% and 8% in the IG^||^ and CG^§^, respectively. The nocturia reports were also reduced 60% and 20% in the IG^||^ and CG^§^, respectively.
Yu, et al. (2011)^30^; Taiwan	EA^†^	To evaluate the effect of EA^†^ on lower urinary tract symptoms in men with BPH^¶^.	n: 37 IG^||^: 18 CG^§^: 19	BPH^¶^ confirmed by transrectal US^§§§^ with I-PSS^††^ score > 8, with no history of prostate surgery; 63.2 (±10.0) years old (IG^||^); 59.8 (±9.0) years old (CG^§^).	AcPts***: Conception Vessels 3 and 4; Stomach 36 and Spleen-Pancreas 6. Needles inserted and handled for 3-5 minutes until reaching Qi. 2 Hz electrostimulation with 2-2.5 mA intensity. 2 sessions/week, 20 min/session.	Needles inserted superficially (subcutaneous tissue), 1 cm lateral to the AcPt*** of the IG^||^, without manual handling or electrostimulation. Same frequency of sessions as in the IG^||^.	1.5 (12 sessions)	I-PSS^††^	Qmax^||||^,Qave^‡‡^ and bladder emptying volume	Changes were not observed in the I-PSS^††^ score between the groups. Increase (improvement) in Qmax^||||^ (*p* = 0.030), Qave^‡‡^ (*p* = 0.026), significant emptying volume (*p* = 0.038) in the IG^||^.
Wang, et al. (2013)^25^; China	EA^†^	To evaluate the effects of EA^†^ on the I-PSS^††^ score, PURV^|||||^, and Qmax^||||^, as well as to explore the differences between AE^†^ in AcPts*** and unrelated points in patients with moderate or severe BPH^¶^.	n: 100 IG^||^: 50 CG^§^: 50	BPH^¶^ determined by I-PSS^††^ score > 8; 64.8 (±7.1) years old (IG^||^); 65.9 (±6.7) years old (CG^§^);	AcPts***: Bladder 33, with manipulation until the patient feels a sensation of weight and numbness. 20 Hz electrostimulation, with maximum intensity tolerated by the patient. 1 session/day completing 5 sessions/week during the 1^st^ and 2^nd^ weeks, and then 3 sessions/week in the 3^rd^ and 4^th^ weeks	Needles inserted at two points approximately 6.7 cm lateral to the AcPts***, without manipulation, with the same electrostimulation as in the intervention group. Same frequency of sessions as in the IG^||^.	1 (16 sessions)	I-PSS^††^	Qmax^||||^ and PURV^||||||^	The I-PSS^††^ score had a 3.2-point higher reduction (improvement) in the IG^||^ than in the CG^§^at week 18 (*p* = 0.001). No changes were observed in Qmax^||||^ or PURV^|||||^.

*AUASI = American Urological Association Symptom Index; ^†^EA = Allethroacupuncture; ^‡^Ph = Phytotherapy; ^§^CG = Control Group; ^||^IG = Intervention Group; ^¶^BPH = Benign Prostate Hyperplasia; **CI = Confidence Interval; ^††^I-PSS = International Prostate Symptom Score; ^‡‡^Qave =Average Urinary Flow Rate; ^§§^PSA = Prostate-Specific Antigen; ^||||^Qmax = Peak Urinary Flow Rate; ^¶¶^CTs = Complementary Therapies; ***AcPts = Acupuncture Points; ^†††^TURP = Transurethral Resection of the Prostate; ^‡‡‡^LUTS = Lower Urinary Tract Symptoms; ^§§^US = Ultrasound; ^||||||^PURV = Post-Urination Residual Volume.

A total of 1,503 men were evaluated in the studies included in this review, of which 1,294 were part of the phytotherapy clinical trials and 209 took part in the electroacupuncture clinical trials. Considering the studies individually, this number varied from 30^20^ to 357 participants^31^. The mean age of the participants was 60.7 years old (±5.6). The follow-up time varied from two to 18 months for the phytotherapy clinical trials (±5.1) and from one to three months for electroacupuncture (±4.7).

To evaluate effectiveness of the CTs, the studies used subjective and objective parameters. The American Urological Association Symptom Index (AUASI)^31^ and the International Prostate Symptom Score (I-PSS)^32^ questionnaires were considered, which subjectively classify LUTS severity as mild (0-7 points), moderate (8-19 points) or severe conditions (20-35 points)^32^. As objective parameters, the following urodynamic outcomes were used: urination frequency, nocturia, peak urinary flow rate (Qmax), average urinary flow rate (Qave), post-urination residual volume (PURV), prostate volume and bladder emptying volume.

Among the studies included in this review, two used the AUASI questionnaire^21^ ^-^ ^22^ and ten resorted to I-PSS for evaluating LUTS^19^ ^-^ ^20^ ^,^ ^23^ ^-^ ^30^. Only one study^20^ did not include any of the urodynamics parameters as one of the outcomes of LUTS evaluation. The majority (n=7) considered at least Qmax and PURV^21^ ^-^ ^25^ ^,^ ^27^ ^-^ ^28^. 

Four phytotherapy studies evaluated the effectiveness of *Saw palmetto* (*S. palmetto*) for controlling LUTS. This herbal medication was compared to placebo at daily doses of 320 mg^19^ ^,^ ^21^ ^-^ ^22^ ^,^ ^26^, 640 mg^22^ and 960 mg^22^. The 320 mg daily dose, fractionated twice a day, significantly improved (*p* < 0.001) the I-PSS score after 24 weeks of treatment^26^ and showed a mean reduction of 4.4 (±5.9) points in the intervention group, with a significant difference (*p* = 0.038) when compared to the placebo after six months^19^. This same dose also reduced 0.68 (±0.35) points in the mean AUASI score of the intervention group, but without a statistically significant difference with the placebo group (95% CI: -0.93 to 1.01)^21^. Higher daily doses of 640 mg and 960 mg reduced 2.20 points (95% CI: -3.04 to -0.36 points) in the mean AUASI score, but an improvement was also observed in the placebo group^22^. None of the evaluated doses of *S. palmetto* improved the Qmax, PURV and prostate volume urodynamics parameters^19^ ^,^ ^21^ ^-^ ^22^ ^,^ ^26^.

Another two clinical trials evaluated effectiveness of the *Ganoderma lucidum* (*G. lucidum)*) herbal medication^23^ ^-^ ^24^. This herbal medication was evaluated at daily doses of 0.6 mg^23^, 6 mg^23^ ^-^ ^24^ and 60 mg^23^. When compared to placebo, daily doses of 6 mg^23^ ^-^ ^24^ and 60 mg^24^ significantly reduced (*p* < 0.001; *p* = 0.012; respectively) the I-PSS scores and slightly improved Qmax and Qave^23^ ^-^ ^24^, but without significant differences between the groups.

In addition, two phytotherapy studies evaluated the effectiveness of using cranberry in LUTS controls^27^ ^-^ ^28^. This herbal medication was compared to placebo at daily doses of 250 mg^28^, 500 mg^28^ and 1,500 mg^27^. At the highest dose of 1,500 mg, fractionated in three times a day, there was a significant reduction (*p* < 0.050) in the I-PSS score of the intervention group, as well as an improvement of the urodynamic parameters, Qmax, Qave and PURV, in 70% of the participants of this group^27^. In the study that evaluated the 500 mg and 250 mg doses there was a reduction of 4.1 (±1.9) and 3.1 (±3.0) points in the I-PSS scores, respectively^28^. The Qmax (*p* = 0.018), Qave (*p* = 0.040), PURV (*p* = 0.027) and bladder emptying volume (*p* = 0.014) urodynamics parameters also indicated significant improvements in the group that received 500 mg of cranberry^28^. 

Regarding the studies that evaluated the effectiveness of electroacupuncture, three included acupuncture points (AcPts) belonging to the bladder meridian^20^ ^,^ ^25^ ^,^ ^29^. Point B32, which belongs to this meridian, was the most used AcPt^20^ ^,^ ^29^. It is noteworthy that, when compared to the conventional medication (Oxybutynin 5 mg) and the placebo tablet, 5 to 10 Hz electrostimulation at the highest tolerated intensity of AcPt B32, promoted LUTS control with a significant improvement of the I-PSS score (*p* < 0.001) and reductions in the urination frequency and nocturia of 20% and 60%, respectively^29^. 

Other AcPts of the bladder meridian were B10 and B40^20^, B21 and B23^29^ and B33^25^. AcPt B33 was the only one that presented a 3.2-point reduction in the I-PSS score of the intervention group, showing a significant improvement (*p* = 0.001) when compared to placebo electroacupuncture when stimulated at 20 Hz at the highest intensity tolerated^25^. 

Another electroacupuncture study evaluated the effectiveness of the AcPts belonging to the different spleen-pancreas (BP6), stomach (E36) and conception vessel (CV3 and CV4) meridians^30^. When stimulated with 3 Hz and intensity of 2 to 2.5 mA, the points of these meridians showed a significant improvement in the Qmax (*p* = 0.030), Qave (*p* = 0.026) and bladder emptying volume urodynamics parameters (*p* = 0.038), when compared to placebo electroacupuncture^30^. However, there was no reduction in the I-PSS score^30^.

The studies included in this review were submitted to methodological quality analysis, based *on the Joanna Briggs Institute (JBI*) Critical Appraisal Tool - Checklist for randomized clinical trials^18^. In this evaluation, six studies were classified as withlow risk of bias, five of which were on phytotherapy^19^ ^,^ ^21^ ^-^ ^24^ and another one on electroacupuncture^25^. 

Three studies were classified as with moderate risk of bias, one on phytotherapy^28^ and the other two on electroacupuncture^20^ ^,^ ^30^. The studies were classified as with moderate risk of bias because they did not describe the losses that occurred during the follow-up or the blinding of the research team responsible for analyzing the outcomes^20^ ^,^ ^28^ ^,^ ^30^. In the phytotherapy study^28^, the method used to blind the researchers who applied the intervention was not informed. In the electroacupuncture studies, failures were observed in the description of the participants’ allocation process^20^, in addition to the absence of double-blinding^20^ ^,^ ^30^. 

High risk of bias was identified in three studies, two on phytotherapy^26^ ^-^ ^27^ and another one on electroacupuncture^29^. As items of methodological weaknesses we have the incomplete description of the randomization method, the differences between the groups at the beginning of the study, and non-description of the blinding and of the sample losses during follow-up^26^ ^-^ ^27^ ^,^ ^29^. The results of the risk assessment in the studies included are presented in Figure 4.

**Figure 4:** Classification of the risk of bias of the studies included (N=12)

Author, year	Q1^||^	Q2^||^	Q3^||^	Q4^||^	Q5^||^	Q6^||^	Q7^||^	Q8^||^	Q9^||^	Q10^||^	Q11^||^	Q12^||^	Q13^||^	Total	Risk of bias
Gerber, et al., 2001^19^	Y*	Y*	Y*	Y*	Y*	U^‡^	Y*	N^†^	N^†^	Y*	Y*	Y*	Y*	76.92%	Low
Bent, et al., 2006^21^	Y*	Y*	Y*	Y*	Y*	Y*	Y*	Y*	Y*	Y*	Y*	Y*	Y*	100%	Low
Barry, et al., 2011^22^	Y*	Y*	Y*	Y*	Y*	Y*	Y*	Y*	Y*	Y*	Y*	Y*	Y*	100%	Low
Ye, et al., 2019^26^	U^‡^	U^‡^	N^†^	U^‡^	U^‡^	U^‡^	Y*	N^†^	Y*	Y*	Y*	Y*	Y*	46.15%	High
Noguchi, et al., 2008^23^	Y*	Y*	Y*	Y*	Y*	N^†^	Y*	Y*	Y*	Y*	Y*	Y*	Y*	92.31%	Low
Noguchi, et al., 2008^24^	Y*	Y*	Y*	Y*	Y*	N^†^	Y*	Y*	Y*	Y*	Y*	Y*	Y*	92.31%	Low
Vidlar, et al., 2010^27^	U^‡^	U^‡^	N^†^	U^‡^	U^‡^	U^‡^	Y*	N^†^	N^†^	Y*	Y*	Y*	Y*	38.46%	High
Vidlar, et al., 2015^28^	Y*	Y*	Y*	Y*	U^‡^	U^‡^	Y*	N^†^	N^†^	Y*	Y*	Y*	Y*	69.23%	Moderate
Johnstone, et al., 2003^20^	Y*	U^‡^	Y*	NA^§^	NA^§^	U^‡^	Y*	N^†^	N^†^	Y*	Y*	Y*	Y*	53.85%	Moderate
Ricci, et al., 2004^29^	U^‡^	U^‡^	N^†^	NA^§^	NA^§^	U^‡^	Y*	N^†^	N^†^	Y*	Y*	Y*	Y*	38.46%	High
Yu, et al., 2011^30^	Y*	Y*	Y*	Y*	NA^§^	U^‡^	Y*	N^†^	N^†^	Y*	Y*	Y*	Y*	69.23%	Moderate
Wang, et al., 2013^25^	Y*	Y*	Y*	Y*	NA^§^	Y*	Y*	Y*	Y*	Y*	Y*	Y*	Y*	92.31%	Low

*Y = yES; ^†^N = No; ^‡^U = Uncertain; ^§^NA = Not Applicable; ^||^Q = Question.

## Discussion

This systematic review sought to identify and evaluate effectiveness of the CTs used for LUTS control in the male population. Among the 12 clinical trials included, eight addressed the use of phytotherapy^19^ ^,^ ^21^ ^-^ ^24^ ^,^ ^26^ ^-^ ^28^ and four dealt with electroacupuncture ^20^ ^,^ ^25^ ^,^ ^29^ ^-^ ^30^. 

For the analysis of the effectiveness of the CTs, one of the methods adopted considered the subjective evaluation of LUTS. Thus, self-administered and internationally validated questionnaires^31^ ^-^ ^32^ were used to classify and standardize the recording of LUTS, being an important tool to determine the severity of this involvement^31^ ^-^ ^34^.

The following stand out among the questionnaires for LUTS evaluation: AUASI questionnaires of the American Urological Association committee^31^ and the I-PSS questionnaire^32^, which refers to an adaptation of AUASI with inclusion of an item that evaluates quality of life by classifying the impact of the discomfort caused by the LUTS on a scale from zero to six^33^. The two instruments assess LUTS severity based on seven questions related to the following: sensation of incomplete bladder emptying, frequent urination, intermittent flow, weak flow, pain or difficulty while urinating, nocturia and urgency^32^. The frequency of each symptom is attributed a score from zero to five, whose sum determines the severity (mild: 0-7 points, moderate: 8-19 points or severe: 20-35 points)^31^ ^-^ ^32^. 

Another method for LUTS evaluation presented by the studies was based on the urodynamic study. This is an objective test to evaluate the function of the lower urinary tract considered as the gold standard in the clinical practice context^35^. Among the urodynamic study parameters considered by the studies, the peak rate of urinary flow (Qmax), prostate volume and post-urination residual volume (PURV) predominated. It is emphasized that only one study did not consider the effectiveness of the intervention applied from the urodynamic evaluation^20^.

As for effectiveness of the CTs, nine studies pointed them as an effective alternative for LUTS control in the male population^19^ ^,^ ^23^ ^-^ ^30^. Phytotherapy stands out, pointed out as one of the most used CTs by the general population^16^ ^,^ ^36^. Phytotherapy is based on the use of medicinal plants for the treatment of certain symptoms, being a practice widely recognized and disseminated by the World Health Organization^37^. The following herbal medications were analyzed in this review: *S. palmetto* ^19^ ^,^ ^21^ ^-^ ^22^ ^,^ ^26^, *G. lucidum* ^23^ ^-^ ^24^ and cranberry^27^ ^-^ ^28^.

Among the four studies that evaluated the effectiveness of *S. palmetto* ^19^ ^,^ ^21^ ^-^ ^22^ ^,^ ^26^, half^19^ ^,^ ^26^ concluded that this herbal medication at a dosage of 320 mg a day was effective in controlling LUTS in men. Both studies indicated a statistically significant reduction in the I-PSS scores in the intervention group when compared to the placebo group^19^ ^,^ ^26^. One of them^26^ showed that *S. palmetto* was also able to improve Qmax, corroborating other studies that also point to an improvement in Qmax, in addition to a reduction in nocturia^12^ ^,^ ^38^.

*S. palmetto*, scientific name *Serenoa repens*, is a herbal medication of the palm family that, due to its anti-inflammatory and anti-androgenic properties, has been commonly used for LUTS control, especially those associated with BPH^39^ ^-^ ^40^. Despite diverse evidence favorable to its use, its applicability and effectiveness in the clinical practice are still questioned^15^. In part, the significant variability in the components’ concentration and bioavailability, depending on the laboratory responsible for the production of the extract, may justify the difficulty defining its effectiveness^41^. In addition, the absence of standardization of the concentrations makes it difficult to establish comparisons between the clinical trials^42^. 

In relation to two studies that did not verify the effectiveness of the *S. palmetto* herbal medication effective, variables such as the type of extract^21^ and the dosage administered^22^ can justify the results obtained. There is more than one type of *S. palmetto* extract, and the forms of ethanolic and hexane extraction present greater clinical effectiveness of the compound^42^. In this context, it is emphasized that one of the studies^21^ adopted the carbon dioxide extract, whose effectiveness is lower^42^. None of the other studies specified the type of *S. palmetto* extract evaluated^19^ ^,^ ^22^ ^,^ ^26^.

Regarding dosage of the *S. palmetto* herbal medication, one of the studies included^22^ concluded that *S. palmetto* was not superior to placebo. This clinical trial^22^ evaluated the effectiveness of *S. palmetto* at staggered doses of 320 mg, 640 mg and 960 mg a day, that is, it considered the double and triple of the dose used in the other studies^19^ ^,^ ^21^ ^,^ ^26^. Thus, considering the discrepancy between the dosages established, a number of precautions are suggested for the interpretation of the results and the relevance of future studies. 

*G. lucidum*, the herbal medication of choice in another two studies of this review^23^ ^-^ ^24^, consists of a type of mushroom that is well-known in Asian countries, with triterpenes and polysaccharides as its outstanding bioactive components^43^ ^-^ ^44^. Although the mechanisms that justify its antitumor, antioxidant and antibacterial effects are not completely elucidated^44^, the satisfactory results presented by the studies included^23^ ^-^ ^24^ are noteworthy, in which there was a significant improvement in the I-PSS scores in the intervention group. Thus, the diverse evidence^23^ ^-^ ^24^ suggests effectiveness of *G*.*lucidum* at a dosage of 6 mg a day for LUTS control in men.

This review also includes two studies^27^ ^-^ ^28^ that analyzed cranberry, scientific name *Vaccinium spp.*, a fruit widely consumed in North American countries to control lower urinary tract infections^45^. The cranberry powder analyzed in both studies was provided by the same laboratory, which favors comparison of the results. At dosages of 250 mg^28^ and 1,500 mg a day^27^, this herbal medication significantly reduced the I-PSS score in the intervention group^27^ ^-^ ^28^. However, considering the urodynamic evaluation, more effective results were better at higher dosages, for example, 1,500 mg a day^27^. It is suggested that the sialic acid found in cranberry extract has anti-inflammatory and analgesic effects, especially by the ability to decrease adhesion of microorganisms in the bladder wall^45^.

Electroacupuncture was another CT evaluated in four clinical trials of this review^20^ ^,^ ^25^ ^,^ ^29^ ^-^ ^30^. The therapeutic effects of acupuncture are obtained from activation of the energy flow or *Qi*, through the insertion of needles in certain AcPts with the objective of restoring homeostatic balance^46^. In this context, electroacupuncture represents an acupuncture variation in which an electric current is applied to the needles seeking to accentuate and enhance the therapeutic effects^47^. The flow of electric current through a biological conductive medium triggers physiological effects, involving electrochemical, electrophysical and electrothermal phenomena. Stimulatory frequency stands out among the most relevant and studied physical parameters in electroacupuncture, especially its relationship with the release of endogenous opioids in analgesic and anti-inflammatory processes^48^. 

As for blinding, the placebo electroacupuncture methods employed in the studies included were as follows: use of points not associated with the AcPts^20^ ^,^ ^25^ ^,^ ^30^, more superficial depth^30^ and absence of electrostimulation^20^ ^,^ ^30^. Due to the blinding difficulty of the clinical studies in this area^47^, it is believed that this fact may justify the uni-blind design of the four studies that evaluated the effect of this therapy^20^ ^,^ ^25^ ^,^ ^29^ ^-^ ^30^.

As for the AcPts used in electroacupuncture, the majority included at least one point referring to the bladder meridian^20^ ^,^ ^25^ ^,^ ^29^, with emphasis on AcPt B32 (*Ciliao*)^20^ ^,^ ^29^. Recent studies have identified significant results for the treatment of BPH symptoms in men^49^ and overactive bladder (OB) symptoms in rats based on AcPt B32 stimulation^50^. It is known that AcPt B32 is one of the four points located in the four sacral foramina, being considered the most important because it has broad indications (voiding dysfunctions, dysmenorrhea, low back pain and sciatica and infertility) and is one of the points that produces the greatest tonifying effect of the Kidney and Essence^51^.

Only one study included in this review chose to use AcPts from other meridians that do not match the bladder’s^30^, namely: spleen-pancreas (BP6 - *Sanyinjiao*), stomach (E36 - *Zusanli*) and conception vessels (CV3 - *Zhongji*; CV4 - *Guanyuan*). A study conducted in rats with overactive bladders evidenced that point B33 (*Zhongliao*) presented a superior effect to points BP6 (*Sanyinjiao*) and B40 (*Weizhong*) with regard to the increase in the interval between the contractions^52^. Therefore, it is suggested that this fact may justify the predominance of protocols that adopt AcPts associated with the bladder meridian when compared to the others. 

Regarding the heterogeneity of the inclusion criteria established by the studies, among the phytotherapy clinical trials, three considered I-PSS or AUASI scores above eight for inclusion of the participants^19^ ^,^ ^21^ ^,^ ^28^, two defined a maximum score of 19 in the I-PSS score^24^ ^,^ ^26^, one defined a maximum limit of 24 points in the AUASI score^22^, and another study considered a minimum score of five in the I-PSS score^23^. Only one study^27^ did not consider the score in the LUTS assessment questionnaires to define the sample. 

In addition, two phytotherapy clinical trials used the AUASI and I-PSS scores to define the BPH diagnosis among their participants^21^ ^,^ ^26^ and the majority only considered the participation of men without prostate surgical history^19^ ^,^ ^21^ ^-^ ^24^ ^,^ ^28^. This heterogeneity to define the LUTS underlying cause and severity can influence evaluation of the effectiveness of the interventions, as it is not defined in the literature what the influence of LUTS severity is in the response to the CTs. 

Similarly, the participants of the electroacupuncture clinical trials presented different selection characteristics. Three studies considered men with I-PSS scores above eight points^20^ ^,^ ^25^ ^,^ ^30^ and one study did not consider any score for sample definition^29^. In relation to the underlying cause for the LUTS, two studies included men with BPH in their samples, one study defined the diagnosis based on the I-PSS score^25^, and another made the diagnosis from a transrectal ultrasound exam^30^. In addition to that, one of the electroacupuncture studies included men who have already undergone transurethral resection of the prostate^29^, while another study^30^ had a sample with only men with no prostate surgical history. 

Another relevant fact refers to the difference in the follow-up time in the studies, which varied between two and 18 months for phytotherapy and one to three months for electroacupuncture. One of the major challenges in conducting clinical studies that assess CT effectiveness is based on the difficulty establishing fixed treatment protocols. It is known that this method is opposed to the basic precepts of the vast majority of the CTs. However, it should be considered that standardization of the ideal follow-up time based clinical studies may favor replicability of the protocols and achieve the same results in future research studies. 

As for the perspectives of including these therapies in Nursing care, it is known that the Nursing Interventions Classification (NIC) includes the “phytotherapy” (2420) and “cutaneous stimulation” (1340) interventions^53^. Nurses are prominent professionals in the implementation and use of several CTs, as the principles of their training are similar to the paradigms of the medical rationalities that involve Integrative Medicine. However, the number of these professionals who work with these therapies or who have the knowledge to prescribe and refer users to this type of care is still reduced. There is a movement, albeit incipient, of nurses who seek specialization courses in this area, which contributes to the dissemination of these therapies to the community, with the consequent improvement of Nursing care^54^.

The selection of clinical trial studies stands out as a limitation of this review. Thus, future expansion is suggested considering different methodological designs. Another limitation was based on the diverse evidence identified and that could not be included due to the effect of the CTs being associated with other conventional treatments such as medical or surgical. As a result, the diverse evidence presented should be considered preliminary, as hypothesis-generating, and as a resource to guide future research studies based on the knowledge gaps identified.

## Conclusion

This systematic review identified and evaluated twelve clinical trials that analyzed the effectiveness of CTS for LUTS control in men. Most of these studies evaluated phytotherapy, which was indicated as an effective alternative in six of the eight clinical trials, as it reduced LUTS frequency from the reduction of the I-PSS scores and urodynamic parameters. We also emphasize that, among the phytotherapy studies, there was predominance of those classified as with low risk of bias. Thus, considering the effectiveness pointed out by half of the studies and their good methodological quality, the indication of phytotherapy for LUTS control in men is supported.

With regard to electroacupuncture, despite the promising results, the suggestion is to develop more robust research studies following methodologies with higher levels of evidence, as only one of the clinical trials was classified as with low risk of bias. It is known that this fact can exert an impact on the effectiveness of the therapy implemented.

In the LUTS context, non-treatment of mild cases or conventional treatment based on medication and surgeries for refractory cases are still predominant alternatives. However, considering the possible effects of the CTs, especially with regard to LUTS control, it becomes fundamental to carry out future research studies capable of generating more consistent recommendations. In general, it is known that CTs are minimally invasive, which implies a lower risk of sequelae when compared to surgical procedures, in addition to having few reports of adverse events, unlike medications. 

## References

[1] D'Ancona C, Haylen B, Oelke M, Abranches-Monteiro L, Arnold E, Goldman H (2019). The International Continence Society (ICS) report on the terminology for adult male lower urinary tract and pelvic floor symptoms and dysfunction. Neurourol Urodyn.

[2] Cameron AP, Lewicky-Gaupp C, Smith AR, Helfand BT, Gore JL, Clemens JQ (2018). Baseline lower urinary tract symptoms in patients enrolled in lurn: a prospective, observational cohort study. J Urol.

[3] Rohrmann S, Katzke V, Kaaks R (2016). Prevalence and progression of lower urinary tract symptoms in aging population. Urology.

[4] Liu SP, Chuang YC, Sumarsono B, Chang HC (2019). The prevalence and bother of lower urinary tract symptoms in men and women aged 40 years or over in Taiwan. J Formos Med Assoc.

[5] Mourad S, Shokeir A, Ayoub N, Ibrahim M, Reynolds N, Donde S (2019). Prevalence and impact of lower urinary tract symptoms: results of the Epic Survey in Turkey. Neurourol Urodyn.

[6] Liao L, Chuang YC, Liu SP, Lee KS, Yoo TK, Chu R (2019). Effect of lower urinary tract symptoms on the quality of life and sexual function of males in China, Taiwan and South Korea: sub-group analysis of a cross-sectional, population-based study. Low Urin Tract Symptoms.

[7] Rhee SJ, Kim EY, Kim SW, Kim SH, Lee HJ, Yoon DH (2019). Longitudinal study of the relationship between lower urinary tract symptoms and depressive symptoms. J Psychosom Res.

[8] Song Q, Abrams P, Sun Y (2019). Beyond prostate, beyond surgery and beyond urology: the "3bs" of managing non-neurogenic male lower urinary tract symptoms. Asian J Urol.

[9] Lokeshwar SD, Harper BT, Webb E, Jordan A, Dykes TA, Neal DE (2019). Epidemiology and treatment modalities for the management of benign prostatic hyperplasia. Transl Androl Urol.

[10] Bechis SK, Kim MM, Wintner A, Kreydin EI (2015). Differential response to medical therapy for male lower urinary tract symptoms. Curr Bladder Dysfunct Rep.

[11] World Health Organization (2013). WHO Traditional medicine strategy: 2014-2023.

[12] Novara G, Giannarini G, Alcaraz A, Cózar-Omo JM, Descazeaud A, Montorsi F (2016). Efficacy and safety of hexanic lipidosterolic extract of Serenoa repens (Permixon) in the treatment of lower urinary tract symptoms due to benign prostatic hyperplasia: systematic review and meta-analysis of randomized controlled trials. Eur Urol Focus.

[13] Zhang W, Ma L, Bauer BA, Liu Z, Lu Y (2017). Acupuncture for benign prostatic hyperplasia: a systematic review and meta-analysis. PLoS One.

[14] Fusco F, Creta M, Nunzio C, Gacci M, Marzi VL, Agro EF (2018). Alpha-1 adrenergic antagonists, 5-alpha reductase inhibitors, phosphodiesterase type 5 inhibitors, and phytotherapic compounds in men with lower urinary tract symptoms suggestive of benign prostatic obstruction: a systematic review and meta-analysis of urodynamic studies. Neurourol Urodyn.

[15] Russo GI, Scandura C, Mauro M, Cacciamani G, Albersen M, Hatzichristodoulou G (2021). Clinical efficacy of Serenoa repens versus placebo versus alpha-blockers for the treatment of lower urinary tract symptoms/benign prostatic enlargement: a systematic review and network meta-analysis of randomized placebo-controlled clinical trials. Eur Urol Focus.

[16] Balneaves LG, Watling CZ, Hayward EN, Ross B, Taylor-Brown J, Porcino A (2022). Addressing Complementary and Alternative Medicine Use Among Individuals with Cancer: An Integrative Review and Clinical Practice Guideline. J Natl Cancer Inst.

[17] Page MJ, McKenzie JE, Bossuyt PM, Boutron I, Hoffmann TC, Mulrow CD (2021). The PRISMA 2020 statement: an updated guideline for reporting systematic reviews. BMJ.

[18] Joanna Briggs Institute (2017). The Joanna Briggs Institute critical appraisal tools for use in JBI systematic reviews: checklist for randomized controlled trials.

[19] Gerber GS, Kuznetsov D, Johnson BC, Burstein JD (2001). Randomized, double-blind, placebo-controlled trial of Saw Palmetto in men with lower urinary tract symptom. Urology.

[20] Johnstone PA, Bloom TL, Niemtzow RC, Crain D, Riffenburgh RH, Amling CL (2003). A prospective, randomized pilot trial of acupuncture of the kidney-bladder distinct meridian for lower urinary tract symptoms. J Urol.

[21] Bent S, Kane C, Shinohara K, Neuhaus J, Hudes ES, Goldberg H (2006). Saw Palmetto for benign prostatic hyperplasia. N Eng J Med.

[22] Barry MJ, Meleth S, Lee JY, Kreder KJ, Avins AL, Nickel JC (2011). Effect of increasing doses of saw palmetto extract on lower urinary tract symptoms: a randomized trial. JAMA.

[23] Noguchi M, Kakuma T, Tomiyasu K, Kurita Y, Kukihara H, Konishi K (2008). Effect of an extract of Ganoderma lucidum in men with lower urinary tract symptoms: a double-blind, placebo-controlled randomized and dose-raging study. Asian J Androl.

[24] Noguchi M, Kakuma T, Tomiyasu K, Yamada A, Itoh K, Konishi F (2008). Randomized clinical trial of an ethanol extract of Ganoderma lucidum in men with lower urinary tract symptoms. Asian J Androl.

[25] Wang Y, Liu B, Yu J, Wu J, Wang J, Liu Z (2013). Electroacupuncture for Moderate and Severe Benign Prostatic Hyperplasia: A Randomized Controlled Trial. PLoS One.

[26] Ye Z, Huang J, Zhou L, Chen S, Wang Z, Ma L (2019). Efficacy and safety of Serenoa repens extract among patients with benign prostatic hyperplasia in China: a multicenter, randomized, double-blind, placebo-controlled trial. Urology.

[27] Vidlar A, Vostalova J, Ulrichova J, Student V, Stejskal D, Reichenbach R (2010). The effectiveness of dried cranberries (Vaccinium macrocarpon) in men with lower urinary tract symptoms. Br J Nutr.

[28] Vidlar A, Student V, Vostalova J, Fromentin E, Roller M, Simanek V (2016). Cranberry fruit powder (Flowens(tm)) improves lower urinary tract symptoms in men: a double-blind, randomized, placebo-controlled study. World J Urol.

[29] Ricci L, Minardi D, Romoli M, Galosi AB, Muzzonigro G (2004). Acupuncture reflexotherapy in the treatment of sensory urgency that persists after transurethral resection of the prostate: a preliminary report. Neurourol Urodyn.

[30] Yu JS, Shen KH, Chen WC, Her JS, Hsieh CL (2011). Effects of electroacupuncture on benign prostate hyperplasia patients with lower urinary tract symptoms: A single-blinded, randomized controlled trial. Evid Based Complement Alternat Med.

[31] Barry MJ, Fowler FJ, O'Leary MP, Bruskewitz RC, Holtgrewe HL, Mebust WK (2017). The American Urological Association Symptom Index for benign prostatic hyperplasia. J Urol.

[32] Harley SJD, Wittert G, Brook NR, Secombe P, Campbell J, Lockwood C (2017). Identifying predictors of change in the severity of untreated lower urinary tract symptoms in men a systematic review protocol. JBI Database System Rev Implement Rep.

[33] Gravas S, Cornu JN, Gacci M, Gratzke C, Herrmann TRW, Mamoulakis C (2018). EAU guidelines on management of non-neurogenic male lower urinary tract symptoms (LUTS), incl. Benign Prostatic Obstruction (BPO).

[34] Parsons JK, Barry MJ, Dahm P, Gandhi MC, Kaplan SA, Kohler TS (2020). Surgical management of lower urinary tract symptoms attributed to benign prostatic hyperplasia AUA guideline amendment 2020. J Urol.

[35] Rosier PFWM, Schaefer W, Lose G, Goldman HB, Guralnick M, Eustice S (2017). International Continence Society good urodynamic practices and terms 2016: urodynamics, uroflowmetry, cystometry, and pressure-flow study. Neurourol Urodyn.

[36] Boing AC, Santiago PHR, Tesser CD, Furlan IL, Bertoldi AD, Boing AF (2019). Prevalence and associated factors with integrative and complementary practices use in Brazil. Complement Ther Clin Pract.

[37] Falzon CC, Balabanova A (2017). Phytotherapy: An introduction to herbal medicine. Prim Care.

[38] Vela-Navarrete R, Alcaraz A, Rodríguez-Antolín A, López BM, Fernández-Gómez JM, Angulo JC (2018). Efficacy and safety of a hexanic extract of Serenoa repens (Permixon(r)) for the treatment of lower urinary tract symptoms associated with benign prostatic hyperplasia (LUTS/BPH): systematic review and meta-analysis of randomised controlled trials and observational studies. BJU Int.

[39] Latil A, Pétrissans MT, Rouquet J, Robert G, Taille A (2015). Effects of hexanic extract of Serenoa repens (Permixon(r) 160 mg) on inflammation biomarkers in the treatment of lower urinary tract symptoms related to benign prostatic hyperplasia. Prostate.

[40] Gravas S, Samarinas M, Zacharouli K, Karatzas A, Tzortzis V, Koukoulis G (2019). The effect of hexanic extract of Serenoa repens on prostatic inflammation: results from a randomized biopsy study. World J Urol.

[41] Giammarioli S, Boniglia C, Stasio L, Gargiulo R, Mosca M, Carratu B (2019). Phytosterols in supplements containing Serenoa repens: an example of variability of active principles in commercial plant-based products. Nat Prod Res.

[42] Gorne RC, Wegener T, Kelber O, Feistel B, Reichling J (2017). Randomized double-blind controlled clinical trials with herbal preparations of Serenoa repens fruits in treatment of lower urinary tract symptoms: an overview. Wien Med Wochenshr.

[43] Qu L, Li S, Zhou Y, Chen J, Qin X (2017). Anticancer effect of triterpenes from Ganoderma lucidum in human prostate cancer cell. Oncol Lett.

[44] Cor D, Knez Z, Hrncic MK (2018). Antitumour, antimicrobial, antioxidant and antiacetylcholinesterase effect of Ganoderma lucidum terpenoids and polysaccharides: a review. Molecules.

[45] Shaheen G, Akram M, Jabeen F, Shah SMA, Munir N, Daniyal M (2019). Therapeutic potential of medicinal plants for the management of urinary tract infections: a systematic review. Clin Exp Pharmacol Physiol.

[46] Liu BP, Wang YT, Chen S (2016). Effect of acupuncture on clinical symptoms and laboratory indicators for chronic prostatitis/ chronic pelvic pain syndrome: A systematic review and meta-analysis. Int Urol Nephrol.

[47] Chen ZX, Li Y, Zhang XG, Chen S, Yang WT, Zheng XW (2017). Sham eletroacupuncture methods in randomized controlled trials. Sci Rep.

[48] Yamamoto H, Kawada T, Kamiya A, Miyazaki S, Sugimachi M (2011). Involvement of the mechanoreceptors in the sensory mechanisms of manual and electrical acupuncture. Auton Neurosci.

[49] Yuan H, Wei N, Li Y, Yu L, Zhang Y, Ong WL (2019). Effect of depth of electroacupuncture on the IPSS of patients with benign prostatic hyperplasia. Evid Based Complement Alternat Med.

[50] Feng QF, Zhang AD, Xing M, Wang X, Ming SR, Chen YI (2020). Electroacupuncture alleviates bladder overactivity via inhabiting bladder P2X3 receptor. Evid Based Complement Alternat Med.

[51] Maciocia G (2017). The Foundations of Chinese Medicine.

[52] Yang L, Wang Y, Mo Q, Liu Z (2017). A comparative study of electroacupuncture at Zhongliao (BL33) and other acupoints for overactive bladder symptoms. Front Med.

[53] Butcher HK, Bulechek GM, Dochterman JM, Wagner CM (2018). Nursing Intervention Classification.

[54] Azevedo C, Moura CC, Corrêa HP, Mata LRF, Chaves ECL, Chianca TCM (2019). Complementary and integrative therapies in the scope of nursing: legal aspects and academic assistance panorama. Esc Anna Nery.

